# Restrictions of the Extraction Process and Bioactivities of Trachyspermum ammi Seed Extract

**DOI:** 10.21315/mjms2022.29.6.18

**Published:** 2022-12-22

**Authors:** Le Pham Tan Quoc

**Affiliations:** Institute of Biotechnology and Food Technology, Industrial University of Ho Chi Minh City, Ho Chi Minh City, Vietnam

Dear Editor,

I read with interest the manuscript titled ‘Evaluation of antibacterial synergism of methanolic extract of *Dracocephalum kotschyi* and *Trachyspermum ammi*’ by Zarei-Yazdeli et al. (1), in which the authors reported that *T. ammi* seed extract (TASE) inhibited some pathogenic microorganisms such as *Pseudomonas aeruginosa*, *Shigella dysenteriae*, *Escherichia coli* and *Staphylococcus aureus*. The paper stated that seed extract from this plant can be considered as a phytochemical preservative in the food industry in the future. However, whilst there are some studies about TASE, it seems to me that the studies relating to the extraction process, biological effects and chemical components of this material are still insufficient.

*T. ammi* is commonly known as ajwain or ajowan. All parts of this plant can be used in medicine, especially the seeds, which contains carbohydrates, fibre, tannins, glycosides, protein, fat, saponins, flavones and minerals while the essential oil (EO) of the seeds possesses a large amount of precious components, including γ-terpinene, *p*-cymene and thymol. This material has many medical applications, such as antifungal, antioxidant, antimicrobial, antinociceptive, cytotoxic, hypolipidemic, antihypertensive, antispasmodic, broncho-dilating actions, antilithiasis, diuretic, abortifacient, antitussive, nematicidal, anthelmintic and antifilarial (2). Nowadays, to research on the biological effects of *T. ammi* seeds, scientists have to extract the bioactive compounds using hydrodistillation methods to separate the EO and conventional extraction methods to isolate the extract. In this study, we only deal with the extraction process of the *T. ammi* seeds.

As we know, solvent plays an important role in the extraction process. For studies involving the extract of *T. ammi* seeds, only pure solvents were used for the extraction, such as methanol (1); ethanol, methanol, *n*-hexane and ethyl acetate (3); and *n*-hexane, chloroform and methanol (4). Bioactivity in the extract depends significantly on the polarity of the solvent. In these studies, although the polarity level increases from *n*-hexane to methanol, water was not used to form an aqueous solvent system. With the presence of water, there are many choices for different polarity levels; hence, the yield of the extraction process can be improved and the bioactivity of the extract enhanced. Using conventional extraction also wastes time: Rajput et al. (5) used the maceration method to extract chemical components in *T. ammi* seeds for 30 days, and Zarei-Yazdeli et al. (1) also spent nearly 8 h, using the Soxhlet machine to isolate extract. This shows that conventional extraction methods, such as maceration or Soxhlet, are not suitable at this time, because of the long extraction time and high operating costs. Nowadays, there are some modern extraction methods that can be used for extracting bioactive compounds from this material, such as microwave-assisted extraction, ultrasound-assisted extraction, enzyme-assisted extraction, supercritical fluid extraction, etc. These methods are green extraction methods, saving time and producing a high yield; the solute is easily recovered and the solvent can be easily recycled.

In addition, the material to solvent (MS) ratios are different in all studies, ranging from 1/10 to 3/10 (g/mL) (1, 3). The ratios were fixed in these studies, but the MS ratios should be investigated because they strongly affect the diffusion of the solute in the solvent. The optimal MS ratio can reduce the quantity of solvent and improve the extraction yield. The yield of the extraction process was also not recorded in almost all studies.

One of the most exciting things about the *T. ammi* seeds is that its EO or extract inhibits the growth of pathogenic bacteria (*Staphylococcus aureus*, *Bacillus subtilis*, *Bacillus cereus*, *Streptococcus pyogenes*, *Escherichia coli*, *Pseudomonas aeruginosa*, *Shigella dysenteriae, Enterococcus faecalis* and *Salmonella typhi*) (1, 3, 6), whilst no yeast inhibition was observed in the previously reported studies. A few studies have recorded that the EO of *T. ammi* seeds can work against pathogenic fungi, including *Candida*, *Aspergillus*, *Chrysosporium* and *Trichophyton* species (7), although there are very few published studies on the antifungal capacity of TASE; almost all studies focused only on the antifungal capacity for *Candida* and *Aspergillus* species (8, 9). This indicates that there are many new concepts to study in the future, and we also hope that these scientific gaps help us to deeply research the extraction process and anti-microorganism capacity of TASE.

There are no official standards and published reports on the specific chemical compounds (non-volatile compounds) that exist in TASE. In my opinion, non-volatile compounds such as phenolic acids, saponins, alkaloids or flavonoids are also extremely important for the bioactivity of the extract. We can completely determine these compounds by high-performance liquid chromatography (HPLC); however, this method is quite complex and costly and needs many different standard substances to screen and identify the chemical components. Therefore, in my opinion, finding new or precious compounds in TASE by HPLC is very interesting and necessary, as this will help us to clarify the roles of the bioactive compounds.

In general, the bioactivity of TASE strongly depends on the extraction conditions and chemical components. I believe that we still have the space to study some issues related to this material and that the scientific results obtained will be very valuable as they will enhance our knowledge, which can be widely applied in the pharmaceutical and medical fields.
